# Supplementary material for: Can Special Light Glasses Reduce Sleepiness and Improve Sleep of Nightshift Workers? A Placebo-Controlled Explorative Field Study
Source: Clocks Sleep. 2020 May 29;2(2):225–45. doi: 10.3390/clockssleep2020018 (PMC7445845; doi:10.3390/clockssleep2020018)
Supplement: Supplementary file 1 [file clockssleep-02-00018-s001.pdf]

Supplementary Material, Light exposure data from LightWatchers.

Calibration of the LightWatchers.

The obtained light exposure measurements were re-calibrated before analysis, to correct for inaccuracies and variation between devices. Specifically, for each device the directional and linear response were calculated and compared to a reference photometer (Hagner cell E4X). The results show large discrepancies between the devices and the reference meter for the response to simulated daylight of linearly decreasing illuminance, while the directional response corresponded to that of the reference meter. Thus, for each device a linear function was calculated, adjusting the device's measurements in order to match with the measurements of the reference meter. These functions were then used to correct the lightlog measurements obtained in the present study in the case of daylight conditions (i.e., illuminance > 1000 lux and UVA > 0.4 μW/m^2).

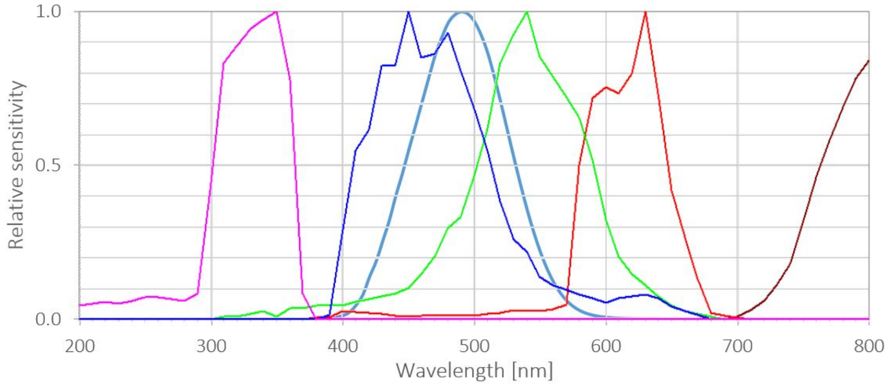

Figure S1 Relative spectral sensitivity of the five sensors (colored lines), action spectrum of melanopic irradiance (light blue dotted line). Pink = UV, Blue= 465nm, Green=540nm, Red=620nm, and brown=875nm.

Table S1. Results of the LightWatchers under treatment conditions

	Illuminance Mean ± *SD*	Mean min. > 10 lux / mean total min.	Mean min. > 1000 lux / mean total min.	UV 350 nm (mW/m^2/nm) Mean ± SD	Bluish sensor 465 nm (mW/m^2/nm) Mean ± SD	Greenish sensor 540 nm (mW/m^2/nm) Mean ± SD	Reddish sensor 620 nm (mW/m^2/nm) Mean ± SD	IR 875 nm (mW/m^2/nm) Mean ± SD
During nightshift (23:15-7:45h)	94±218	352 / 510	1.1 / 510	$3.9\cdot10^{-2}$ ±$1.6\cdot10^{-1}$	$7.6\cdot10^{-1}$±1.7	1.3±2.3	1.1±2.0	$1.7\cdot10^{-1}$ ±$8.5\cdot10^{-1}$
During last hour of nightshift (6:45-7:45h)	132±472	43 / 60	0.7 / 60	$6.7\cdot10^{-2}$ ±$4.1\cdot10^{-1}$	1.2±3.9	1.8±4.3	1.5±4.0	$3.9\cdot10^{-1}$ ±2.3
During 24h before first nightshift	1116±5295	623 / 1440	119 / 1440	1.4±8.1	13±68	14±73	13±69	7.0±39

(23:15-23:15h)								
During 24h after last nightshift (23:15-23:15h)	1150±5270	666 / 1440	125 / 1440	1.6±7.8	13±61	13±66	13±63	7.0±35
During 24h on First Night (23:15-23:15h)	150±1206	614 / 1440	21 / 1440	$1.3\cdot10^{-1}$ ±1.6	1.5±13	1.8±13	1.6±12	$7.0\cdot10^{-1}$ ±6.6
During 24h on Night (23:15-23:15h)	393±2631	690 / 1440	50 / 1440	$4.5\cdot10^{-1}$ ±3.6	4.3±31	4.7±33	4.6±33	2.5±20
During 24h on Last Night (23:15-23:15h)	587±3109	841 / 1440	89 / 1440	$6.9\cdot10^{-1}$ ±3.9	5.9±31	6.4±34	6.2±34	3.3±20
During daytime indoors (6:00-18:00h)	107±333	325 / 720	12 / 720	$7.4\cdot10^{-2}$ $\pm5.4\cdot10^{-1}$	1.5±6.0	1.7±6.4	1.7±6.1	$8.4\cdot10^{-1}$ ±3.7
Between work and bed after nightshift	898±2414	48 / 60	11 / 60	$9.1\cdot10^{-1}$ ±2.9	9.8±24	10±24	8.9±22	4.7±13
Between work and bed after nightshift (adj. for sunglasses)	452±1151	48 / 60	13 / 60	$5.5\cdot10^{-2}$ $\pm3.4\cdot10^{-1}$	1.0±3.4	3.5±7.5	6.4±17	4.7±13
During 2h before bed on normal days	188±1622	46 / 120	1.7 / 120	$1.1\cdot10^{-1}$ $\pm9.2\cdot10^{-1}$	2.1±18	2.6±22	3.3±28	2.0 ±18
During 2h before bed on nightshift days	495±1707	91 / 120	7.7 / 120	$4.6\cdot10^{-1}$ ±2.2	5.3±17	5.7±18	5.1±16	2.4±9.4
During bed on normal days	8.0±55	39 / 480	0.0 / 480	$8.3\cdot10^{-3}$ $\pm5.1\cdot10^{-2}$	$9.6\cdot10^{-2}$ $\pm6.6\cdot10^{-1}$	$1.2\cdot10^{-1}$ $\pm8.1\cdot10^{-1}$	$1.3\cdot10^{-1}$ $\pm8.7\cdot10^{-1}$	$6.1\cdot10^{-2}$ $\pm3.6\cdot10^{-1}$

During bed on nightshift days	4.7±58	45 / 480	0.1 / 480	$6.8 \cdot 10^{-3} \pm 8.1 \cdot 10^{-2}$	$7.3 \cdot 10^{-2} \pm 6.7 \cdot 10^{-1}$	$8.7 \cdot 10^{-2} \pm 7.0 \cdot 10^{-1}$	$1.2 \cdot 10^{-1} \pm 7.7 \cdot 10^{-1}$	$8.1 \cdot 10^{-2} \pm 4.9 \cdot 10^{-1}$
During 2h after bed on normal days	1663±5875	100 / 120	9.0 / 120	2.0±8.4	20±76	21±81	20±75	10±41
During 2h after bed on nightshift days	1896±5995	97 / 120	13 / 120	2.6±9.3	23±79	24±85	24±84	14±49

Table S2 Results of the LightWatchers under placebo conditions

	Illuminance Mean ± *SD*	Mean min. > 10 lux / mean total min.	Mean min. > 1000 lux / mean total min.	UV 350 nm (mW/m2 /nm) Mean ± SD	Bluish sensor 465 nm (mW/m2 /nm) Mean ± SD	Greenish sensor 540 nm (mW/m2 /nm) Mean ± SD	Reddish sensor 620 nm (mW/m2 /nm) Mean ± SD	IR 875 nm (mW/m2 /nm) Mean ± SD
During nightshift (23:15-7:45h)	74±163	334 / 510	0.8 / 510	$4.9 \cdot 10^{-2} \pm 1.8 \cdot 10^{-1}$	$6.9 \cdot 10^{-1} \pm 1.5$	1.2±2.0	$9.5 \cdot 10^{-1} \pm 1.6$	$1.4 \cdot 10^{-1} \pm 5.5 \cdot 10^{-1}$
During last hour of nightshift (6:45-7:45h)	101±356	40 / 60	0.5 / 60	$7.0 \cdot 10^{-2} \pm 3.6 \cdot 10^{-1}$	1.0±3.3	1.4±3.4	1.2±3.1	$2.9 \cdot 10^{-1} \pm 1.5$
During 24h before first nightshift (23:15-23:15h)	1377±6397	614 / 1440	126 / 1440	2.5±12	17±89	18±93	17±88	10±52
During 24h after last nightshift (23:15-23:15h)	1255±5428	651 / 1440	145 / 1440	7.4±37	14±66	15±70	14±66	2.3±10
During 24h on First Night (23:15-23:15h)	444±2983	677 / 1440	62 / 1440	$7.4 \cdot 10^{-1} \pm 6.1$	5.1±40	5.3±40	4.9±38	2.7±23
During 24h on Night (23:15-23:15h)	165±1224	661 / 1440	27 / 1440	$2.8 \cdot 10^{-1} \pm 2.5$	2.5±24	2.8±25	2.6±25	1.3±15
During 24h on Last Night	624±3564	812 / 1440	82 / 1440	$9.8 \cdot 10^{-1} \pm 6.2$	7.3±47	7.7±49	7.3±47	4.0±27

(23:15-23:15h)								
During daytime indoors (6:00-18:00h)	122±807	322 / 720	12 / 720	$2.3\cdot10^{-1}$ ±1.9	1.8±15	2.1±16	2.0±16	1.0±9.4
Between work and bed after nightshift	1274±4206	49 / 60	10 / 60	1.5 ±6.2	13±45	14±46	12±42	6.4±25
Between work and bed after nightshift (adj. for sunglasses)	839±3242	49 / 60	14 / 60	$6.2\cdot10^{-1}$ ±4.1	5.2±32	7.5±33	9.7±37	6.4±25
During 2h before bed on normal days	71±704	55 / 120	0.8 / 120	$1.4\cdot10^{-1}$ ±1.1	$8.2\cdot10^{-1}$±6.6	$8.9\cdot10^{-1}$±6.8	$9.7\cdot10^{-1}$±6.3	$6.3\cdot10^{-1}$ ±3.7
During 2h before bed on nightshift days	694±2802	91 / 120	6.8 / 120	$7.3\cdot10^{-1}$ ±3.8	7.3±30	7.8±32	7.0±31	3.5±17
During bed on normal days	3.8±32	30 / 480	0.0 / 480	$6.9\cdot10^{-3}$ ±$1.8\cdot10^{-1}$	$4.0\cdot10^{-2}$ ±$3.1\cdot10^{-1}$	$4.4\cdot10^{-2}$ ±$3.4\cdot10^{-1}$	$4.8\cdot10^{-2}$ ±$3.7\cdot10^{-1}$	$2.3\cdot10^{-2}$ ±$2.0\cdot10^{-1}$
During bed on nightshift days	7.8±120	59 / 480	0.3 / 480	$2.0\cdot10^{-2}$ ±$3.0\cdot10^{-1}$	$9.8\cdot10^{-2}$ ±1.2	$1.1\cdot10^{-1}$ ±1.2	$1.3\cdot10^{-1}$ ±1.1	$7.6\cdot10^{-2}$ ±$6.5\cdot10^{-1}$
During 2h after bed on normal days	1047±4321	95 / 120	7.3 / 120	3.0±15	14±67	15±69	14±64	7.3±37
During 2h after bed on nightshift days	1231±4835	89 / 120	9.3 / 120	2.1±9.7	16±71	16±73	15±69	8.5±40
